# An In Vitro Analysis of the Effects of Mouthwashes on the Surface Properties of Composite Resin Restorative Material

**DOI:** 10.7759/cureus.65021

**Published:** 2024-07-21

**Authors:** Rashmi Rekha Mallick, Priyanka Sarangi, Shradha Suman, Subhranshu Sekhar Sahoo, Ayushi Bajoria, Gaurav Sharma

**Affiliations:** 1 Department of Conservative Dentistry and Endodontics, Srirama Chandra Bhanja (SCB) Dental College and Hospital, Cuttack, IND; 2 Department of Pedodontics and Preventive Dentistry, Srirama Chandra Bhanja (SCB) Dental College and Hospital, Cuttack, IND; 3 Department of Public Health Dentistry, Srirama Chandra Bhanja (SCB) Dental College and Hospital, Cuttack, IND

**Keywords:** oral care, surface roughness, microhardness, mouth rinses, composite resin

## Abstract

Background: Restorative composite resins have clinical prime importance in modern dental practice, but numerous factors influence their prognosis in the oral environment. Their interaction with oral care products, like mouthwashes, is one such factor. This study aimed to evaluate the quantifiable effects of different mouthwashes on the surface properties of the composite resin restorative material.

Methodology: The method involved formulating 90 samples of nanohybrid composite resins (Medicept Dental India Private Limited, Mumbai, India). The samples were treated with 1% alcohol, without alcohol, and with a saline solution (control). Surface roughness (Ra values) and microhardness had been checked before and after an exposure period of 24 hours. Data were thus tabulated, and from that, average values of surface roughness and microhardness were derived. This data was analyzed using the IBM SPSS Statistics for Windows, V. 26.0 (IBM Corp., Armonk, NY). Analysis of variance (ANOVA) and a t-test were used to compare the means of the variables. The level of significance was fixed at p < 0.05.

Results: The surface roughness value was significantly highest in the ethanol-containing mouthwash-treated samples after 24 hours of exposure (p < 0.05). The microhardness was statistically lower in these samples (p < 0.05).

Conclusion: These research data give quantitative information, however, about the mass effect of mouthwashes on the composite resin restorative material. The presented changes, which were illustrated by the values of surface roughness and microhardness, are drawing attention to the fact that a cautious approach must be taken in the recommendations for oral care in the provision of intensive treatments with restorative composite resins.

## Introduction

Hence, composite dental materials are of immense importance to the Modern Renaissance within the dentistry domain, for the most part, to aid and hold onto tooth structure, oral function, and aesthetics. The more critical multiple factors that influence restoration longevity include the restorative material applied, the type of cavity characteristics, and interaction with oral care products [1-3]. These materials face even harsher durability challenges in a dynamic oral environment, including the acts of saliva, microbial flora, and interactions with materials from oral care. Among the most famous products used by customers daily in oral care is mouthwash. It makes the oral hygienist choose which mouthwash to apply widely [4]. Generally, normal functions of mouthwashes are aiding health maintenance, prevention against plaque accumulation, and gingivitis control [5,6]. However, their impact on the physical and mechanical properties of the composite resins has been an active area under investigation. Nanohybrid composite resins have gained prominence due to their favorable physical and mechanical attributes, such as high compressive strength, exceptional wear resistance, and superior aesthetic results, rendering them suitable for a broad spectrum of dental restorations [7].

Among other things, one of the primary goals in the practice of dental medicine through composite restoration would be the attainment of a smooth surface, which may be critical not merely in terms of aesthetic appearance but also for the health of the intraoral soft tissues and the marginal interface integrity of restorations. Increased surface roughness can result in discoloration, staining, and loss of gloss of composite resin restoration [7] and may also lead to colonization by oral microorganisms [8]. Therefore, one of the factors that has a significant effect on the composite resin's clinical performance is its surface finish.

Hardness is an essential parameter of material ingredients, characterized by long-term survivability in oral cavities. Hardness is the resistance of materials to indentation or penetration. Microhardness is a measure of both the strength of the material and its approximation of the resistance to wear [9]. Most mouthwashes have active ingredients such as alcohol, antimicrobial agents, and others that react with the material of the composite resin restoration. All these result in changes in surface roughness and microhardness, both potentially compromising features of the underlying composite resin restoration [10-13].

Listerine Original (Johnson & Johnson, Italy) is an alcohol-containing mouthwash known for its antiseptic properties. It contains active ingredients such as essential oils (e.g., eucalyptol, menthol, thymol, and methyl salicylate) that are effective against oral bacteria, helping to reduce plaque buildup and maintain oral hygiene. This mouthwash is widely used for its refreshing taste and ability to provide a clean feeling after use. In the study, Listerine Original was included to evaluate the effects of alcohol-containing mouthwash on the properties of nanohybrid composite resin discs. Clohex Heal (Dr. Reddy's Laboratories Ltd., Hyderabad, India) is an alcohol-free mouthwash formulated with chlorhexidine gluconate as its active ingredient. Chlorhexidine is recognized for its antimicrobial properties, effectively targeting bacteria responsible for plaque formation and gingivitis. Clohex Heal is often recommended for individuals who prefer alcohol-free oral care products or those with sensitivity to alcohol. In the study, Clohex Heal was chosen to assess the impact of an alcohol-free mouthwash on the tested composite resin discs, providing a comparison against the alcohol-containing mouthwash [10-13].

Both mouthwashes were used in the study to simulate their long-term effects equivalent to daily rinsing for two years, allowing researchers to evaluate their potential influences on the surface roughness and microhardness of the composite resin discs under controlled conditions. These products represent common choices in oral care routines, making their evaluation pertinent to understanding their effects on dental materials used in restorative procedures.

Despite the clinical relevance of the action of composite resin restorative material on mouthwashes, the extent of this action is yet to be fully unraveled. The present study evaluated the surface roughness and microhardness values of composite resin incorporated with different compositions of mouthwashes. Post-immersion of the composite resin in various types of solutions, the current research framed a null hypothesis that stated that both the composite resin surface roughness and hardness will not be significantly altered.

## Materials and methods

Ninety nanohybrid composite resin (Medicept Dental India Private Limited, Mumbai, India) discs of equal dimensions (8 × 2 mm) were prepared using plastic molds. The composite material was inserted into the mold with the aid of a Teflon-coated composite filling instrument. After the composite resin was filled into the plastic mold, it was covered with a transparent mylar strip. To get a smooth surface and remove any excess material, a glass plate was placed over it, and light pressure was applied. After the composite resin discs were light-cured for 20 seconds per side using a Bluephase N*M curing unit (Ivoclar Vivadent, Schaan, Liechtenstein), microhardness testing was performed on the surface that had been directly exposed to the curing light. This surface was chosen to assess the hardness properties influenced by the polymerization process. After curing the composite, discs were polished using Soflex discs (3M ESPE, dental products, Seefeld, Germany). The sample size was calculated using G*Power software (Heinrich Heine University Düsseldorf, Düsseldorf, Germany) with an effect size of 0.52, a significance of 5%, and a power of the study of 95%.

Samples were randomly divided into three groups, each consisting of 30 samples. The baseline surface roughness of each specimen was recorded with a Surface Profilometer (S. L. Technologies, Delhi, India). Next, using the Vickers microhardness tester (A. S. I. Sales Private Limited, New Delhi, India), the baseline microhardness of each specimen was noted. The mean Vickers hardness number (VHN) measurements of each surface of the composite resin disc were performed by three randomly performed diamond indentations (2 μm in diameter). A microhardness tester (LG HV 1000; High Technology Laboratory Certificate S.A.C.) was used at 30 N (300 gf) for 10 s20. The formula used to calculate VHN is as follows: HV = 1.854⋅F/d^2 ^where F is the applied force in kgf and d is the average diagonal length in mm. After baseline measurement, the samples were immersed in different mouthwashes and saline solutions.

Immersion of composite specimens in mouthwashes

**Figure 1:**
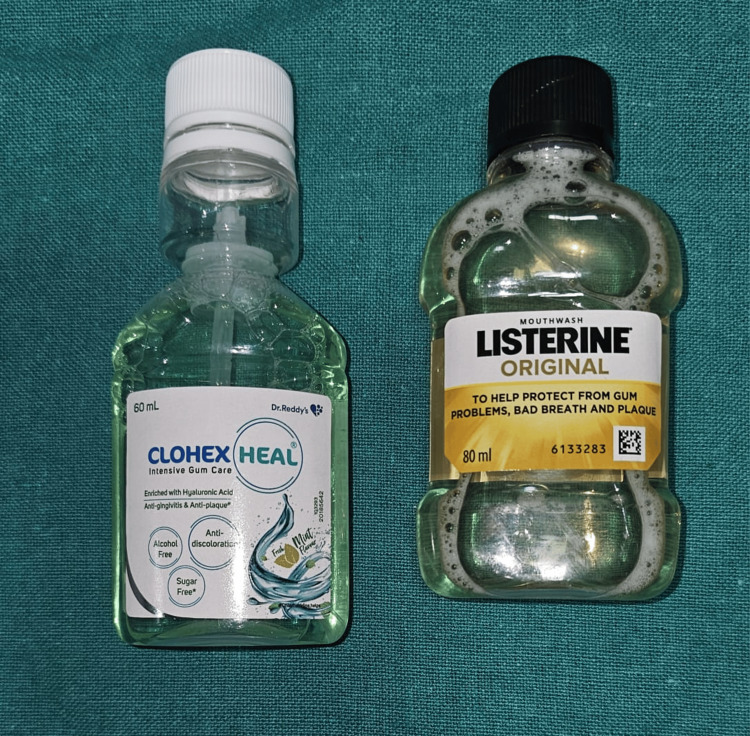
Materials used in the study

**Figure 2:**
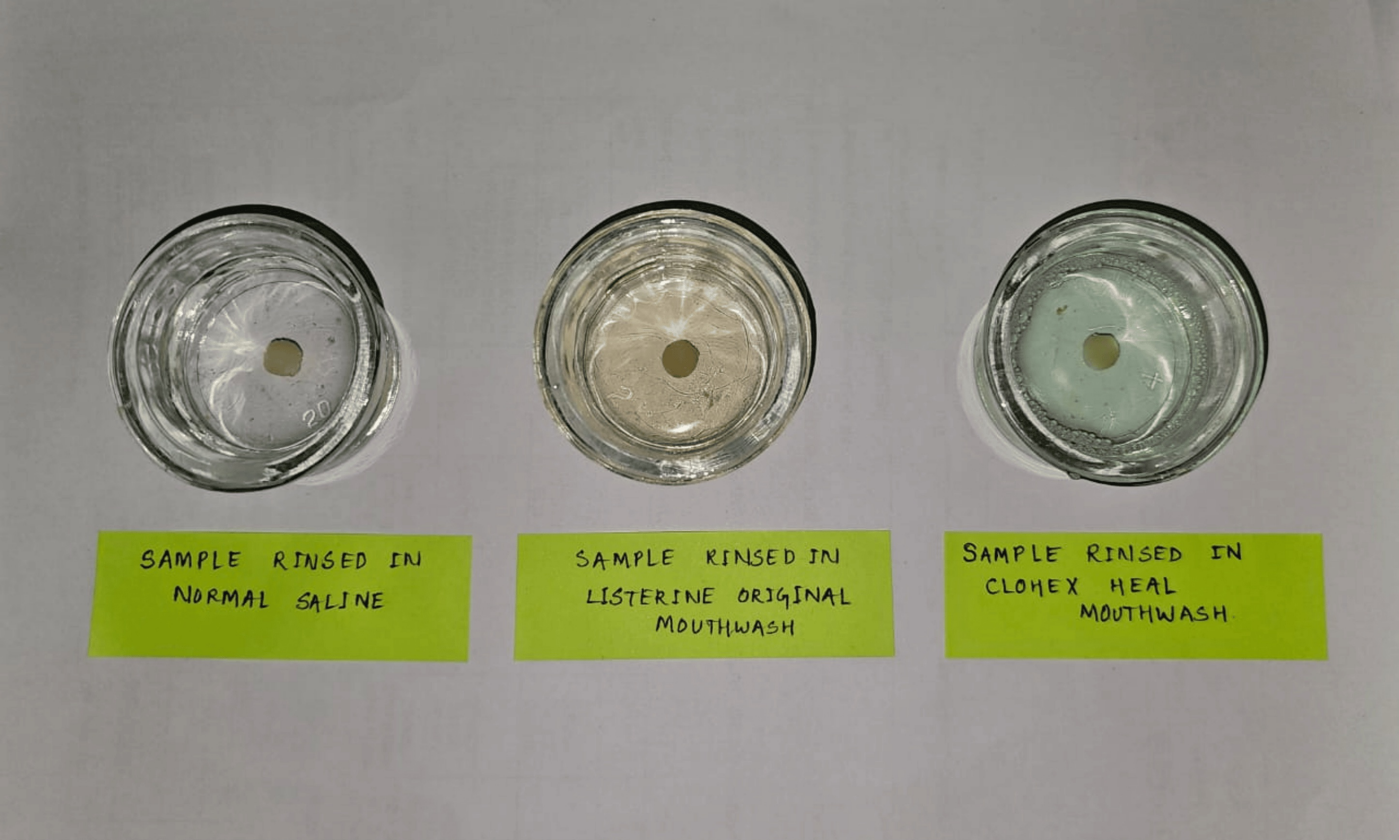
Group-wise immersion of the samples in the solution

For this study, we have taken two readily available mouthwashes: Listerine Original containing alcohol and Clohex Heal, an alcohol-free mouthwash. Almost the equivalent of two minutes of daily rinsing for two years, each specimen was submerged in 20 ml of chosen mouthwashes and regular saline for 24 hours at 37°C in an incubator [14]. In Group A, samples were exposed to Listerine Original, an alcohol-containing mouthwash. In Group B, samples were exposed to Clohex Heal, an alcohol-free mouthwash. In Group C, the control group was exposed to a saline solution (Figure 1, Figure 2).

The samples were removed from mouthwashes and normal saline and left to air dry at room temperature (25°C). To assess the change, measurements of surface roughness and surface microhardness were made again. The experiments were conducted by trained dental professionals. Operators were trained in the use of specific instruments and methodologies to ensure consistency and reliability of the results.

Statistical analysis

The IBM SPSS Statistics for Windows, V. 26.0 (IBM Corp., Armonk, NY) was used for statistical analysis of the data, and MS Excel was used for descriptive statistics. Analysis of variance (ANOVA) and the t-test were used to compare the means of the variables. A p-value of less than 0.05 is regarded as significant.

## Results

The study evaluated the impact of different treatments on surface roughness and microhardness across three groups: Group A, Group B, and Group C. The results are summarized in Table 1 and Table 2, highlighting significant changes in both parameters for Groups A and B compared to the control group (Group C).

**Table 1:** The mean surface roughness differences for Groups A, B, and C Descriptive statistics are presented as mean ± SD. A dependent t-test is used to compare within-group statistics. P represents probability value at a level of significance (p < 0.05) SD: standard deviation

Groups	Pre-mean	Post-mean	F-value	T-value	P-value
Group A	0.21 ± 0.02	0.35 ± 0.03	2.15	3.50	0.001
Group B	0.19 ± 0.02	0.30 ± 0.03	2.10	3.00	0.001
Group C	0.15 ± 0.05	0.21 ± 0.02	2.06	1.50	0.072

**Table 2:** The mean differences in microhardness for Groups A, B, and C Descriptive statistics are presented as mean ± SD. A dependent t-test is used to compare within-group statistics. P represents probability value at a level of significance (p < 0.05) SD: standard deviation

Groups	Pre-mean	Post-mean	F-value	T-value	P-value
Group A	57.92 ± 2.28	57.07 ± 2.39	1.5	2.25	0.011
Group B	57.66 ± 2.29	57.56 ± 2.31	1.3	1.95	0.002
Group C	57.72 ± 2.98	57.71 ± 2.24	1.2	0.75	0.056

The mean surface roughness for Groups A, B, and C were 0.14, 0.11, and 0.06, respectively. Post-treatment, a significant increase in surface roughness was observed in both Group A and Group B. Specifically, Group A showed an increase from 0.21 ± 0.02 to 0.35 ± 0.03 (p = 0.001) and Group B from 0.19 ± 0.02 to 0.30 ± 0.03 (p = 0.001). The control group (Group C) also experienced an increase in surface roughness, from 0.15 ± 0.05 to 0.21 ± 0.02, although this change was not statistically significant (p = 0.072). The F-values for Groups A, B, and C were 2.15, 2.10, and 2.06, respectively, indicating variability in surface roughness changes among the groups. The t-test values for Groups A and B (3.50 and 3.00, respectively) confirmed the significant increase in surface roughness within these groups, while the t-test value for Group C (1.50) supported the non-significant change in the control group (Table 1).

The analysis of microhardness revealed a significant decrease in both Group A and Group B, with mean values of 0.85 and 0.10, respectively, compared to the control group's mean of 0.01. Group A experienced a notable reduction in microhardness, while Group B also showed a significant decline. In contrast, the control group (Group C) exhibited a minor reduction in microhardness, which was not statistically significant (p = 0.056) (Table 2).

These results underscore the differential impact of the treatments on surface roughness and microhardness, with significant changes observed in the treated groups (A and B) compared to the control group (C). The statistical analysis confirms that the treatments administered to Groups A and B significantly altered both surface roughness and microhardness, whereas the control group showed minimal changes that were not statistically significant.

## Discussion

The primary factors driving the demand for composite resin restorative materials worldwide are superior aesthetics and clinical durability. Within the field of restorative dentistry, the continuous advancement of nanotechnology has enabled the challenging accomplishment of developing nano-filled and nanohybrid composite. Nanohybrid composites are very promising for usage as anterior and posterior restorations due to their enhanced mechanical and optical properties [15]. These restorative materials experience several changes within the oral cavity because of masticatory pressures, temperature fluctuations, and frequent use of commercial mouthwashes.

The effects of various types of mouthwash on the composite resin's surface roughness and microhardness were investigated in this research. This study used mouthwashes with and without alcohol since dentists typically recommend both to reduce plaque accumulation and prevent gingivitis. The control group consisted of nanohybrid composite discs immersed in normal saline.

The findings of this investigation rejected the null hypothesis, which held that the various types of mouthwash did not affect the surface roughness and microhardness of the composite resin. The observed increase in surface roughness among all groups suggests that the structural integrity of composite resin may be compromised.

The findings of this investigation demonstrated that compared to alcohol-free Clohex Heal mouthwash, exposure to Listerine Original mouthwash containing alcohol increased the composite resin's surface roughness. It is supported by the findings of previous studies [16,17]. This may be due to the plasticizing effect of alcohol [18]. Additionally, prior research has indicated a clear correlation between the alcohol content of mouthwash and the composite resin's surface roughness [18].

Both alcohol-containing and alcohol-free mouthwashes contain various components that can interact with composite resins. Alcohol and essential oils in alcohol-containing mouthwashes can soften and degrade the resin matrix, while chlorhexidine, fluoride, acids, and humectants in alcohol-free mouthwashes can lead to increased surface roughness, changes in mechanical properties, and degradation of the resin structure [19]. Previous studies also reported increased surface roughness of composite resin exposed to mouthwash containing chlorhexidine [16,20].

The slight increase in surface roughness in the control group prompts consideration of the role of the normal saline solution. Although traditionally considered inert, this finding suggests that even seemingly harmless substances may have subtle effects on composite resin restorative material. While composite resins have inherent surface characteristics, external factors like saline immersion can contribute to alterations in surface roughness; this emphasizes the necessity of conducting thorough research on how different oral care products affect composite resin restoration.

This study demonstrated that the microhardness of composite resin immersed in both types of mouthwash significantly decreased, with the alcohol-containing Listerine Original mouthwash group experiencing a greater reduction in microhardness. This outcome is consistent with previous research highlighting alcohol's ability to impact resin-based materials, potentially lowering their microhardness [21,22]. The study done by Alessa found the negative effect of acidic mouthwashes on the microhardness of composite dental restorations [10]. George and Kavyashree [23] and Kocchar et al. [24] described how the presence of alcohol in Listerine® decreases the hardness of dental restorative materials, while a study done by Gürgan et al. [25] found some extent of the reduction of microhardness even with alcohol-free mouthwashes. 

The reduction in microhardness across all groups is a notable finding with potential implications for the mechanical properties of composite resin. The observed reductions may indicate a vulnerability of the material to chemical interaction with the components of the mouthwash. Alcohol-containing mouthwashes (Listerine Original) may soften or degrade the resin matrix over time by disrupting polymer chains and potentially causing swelling or compositional changes. This can lead to increased surface roughness as the resin becomes more susceptible to wear. In contrast, alcohol-free mouthwashes (Clohex Heal), containing chlorhexidine gluconate, primarily target microbial flora without solvent effects. While chlorhexidine is less likely to degrade the resin, prolonged exposure may still alter surface chemistry and affect properties like microhardness and roughness [10]. 

Both types of mouthwashes aim to control oral microbial flora, which can adhere to and degrade dental materials. Microbes and their metabolic by-products can compromise composite resins over time, leading to surface roughening and potential mechanical property deterioration. Effective reduction in microbial load by mouthwashes may indirectly contribute to preserving the integrity of composite resin surfaces. This underscores the importance of understanding the chemical compatibility of composite resin restorative material with common oral care products. It should be noted that the observed changes in surface roughness and microhardness of composite resin while statistically significant might not necessarily translate into clinically significant outcomes. To find out how changes in surface roughness and microhardness relate to material durability and clinical performance, more research is needed.

While this study provides valuable insights into the effects of different mouthwashes on the surface roughness and microhardness of composite resin restorative materials, several limitations must be acknowledged. Firstly, the in vitro nature of the experiments may not fully capture the dynamic and multifactorial environment of the oral cavity, where factors such as saliva, enzymatic activity, and microbial biofilm play significant roles. The lack of these biological elements may affect the extrapolation of the findings to real-world clinical scenarios. Additionally, the duration of mouthwash exposure in the laboratory setting may not accurately reflect the intermittent and varied use of mouthwashes by patients. The study's findings are also limited by the specific types and formulations of mouthwashes tested, which may not encompass the full spectrum of commercially available products. Future research should consider long-term clinical trials and include a broader range of mouthwashes and composite resin materials to enhance the generalizability and clinical relevance of the results.

## Conclusions

This study provides quantitative information on the substantial impact of mouthwashes on composite resin restorative materials. The results indicated that alcohol-containing mouthwashes significantly increase surface roughness and decrease microhardness compared to alcohol-free mouthwashes and saline solutions. These findings suggest that the chemical composition of mouthwashes can affect the durability and integrity of composite resins used in dental restorations. Therefore, dental professionals need to consider the type of mouthwash recommended to patients, especially those with extensive composite restorations. Choosing appropriate oral care products can help maintain the longevity and aesthetic quality of composite restorations, ultimately contributing to better oral health outcomes. This study highlights the need for further research to explore the long-term effects of various oral care products on different types of dental materials.
